# A New Brace for Maintaining the Neck in a Suitable Position Following Tracheal Reconstruction

**Published:** 2014-05

**Authors:** Bizhan Ziaian, Ali Foroutan, Maryam Tahamtan, Sam Moslemi

**Affiliations:** 1 Department of Thoracic Surgery, Nemazee Hospital, Shiraz University of Medical Sciences, Shiraz, Iran;; 2 Department of General Surgery, Fagihi Hospital, Shiraz University of Medical Sciences, Shiraz, Iran;; 3 Department of Internal Medicine, Nemazee Hospital, Shiraz University of Medical Sciences, Shiraz, Iran

**Keywords:** Brace, Flexion, Position

## Abstract

Segmental resection and end-to-end anastomosis is the treatment of choice for patients suffering from tracheal stenosis for whom conservative management is not planned. A complication of this procedure is tension-induced anastomotic failure. To prevent this complication, maintaining the neck in full flexion by means of a suture between the chin and upper chest is a traditional approach. We have designed a new brace (Shiraz brace) that securely supports the neck in this position and decreases the bothersome use of the suture alone.

## Introduction

Among several therapeutic options for tracheal stenosis, segmental resection and tracheal reconstruction with end-to-end anastomosis is the most effective treatment of choice for patients who cannot be considered for conservative management.^1^ One of the most striking complications of tracheal reconstruction is disruption of anastomosis due to the tension induced by approximation of the divided parts of the trachea. Therefore, an important step in the surgical approach is proper cervical flexion to release tension on the anastomosis which is traditionally obtainable from suturing the chin into the anterior part of the upper chest.^2^^,^^3^ This procedure is not only bothersome but also has the potential for rupture especially during awakening the patient from anesthesia because patients are unaware of the stiches. This procedure does not prevent lateral movement of the neck which can be disastrous, i.e. disruption of the anastomosis. With the purpose of additional support, we report a newly-designed brace (Shiraz brace) that has been applied in our patients.

## Clinical Summary

**Figure 1:**
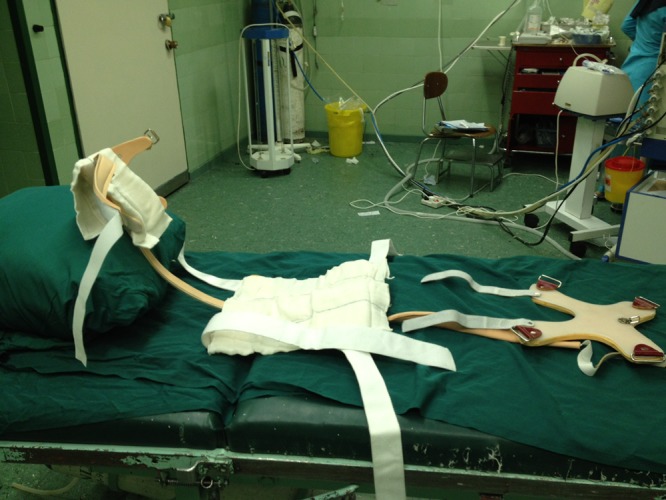
Multiple sections of the Shiraz Brace are demonstrated.

**Figure 2:**
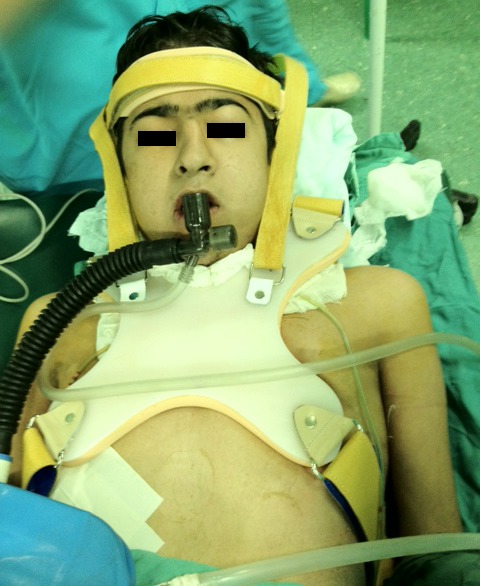
Applied brace (frontal view) in a case of tracheal reconstruction.

**Figure 3:**
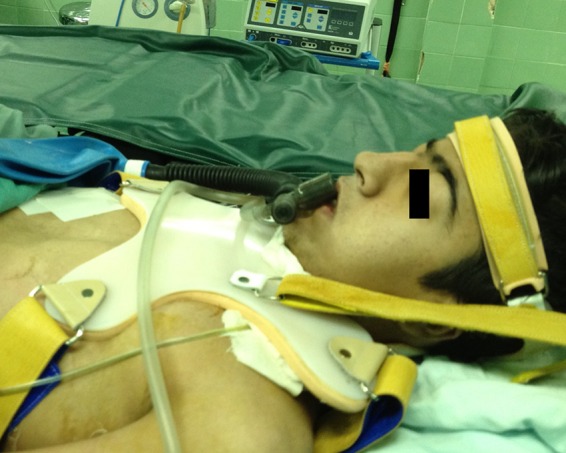
Applied brace (lateral view) in a case of tracheal reconstruction.

In addition to a suture placed between the skin of the chin point and anterior part of the upper chest in the midline position, we have designed a fiberglass-made brace that weighs 800 g (figure 1) with the intent to maintain constant complete neck flexion, post-operatively, in patients who refer to our center for tracheal reconstruction. The brace is 100 cm in length and extends from the occiput to the sacrum. It has the capacity to be tighter or looser and precisely set by strip bands on both shoulders. A butterfly part of this brace is placed anterior to the chest wall to fix the brace onto the trunk. Patients can wear this brace when sleeping, sitting or walking during the first week after reconstruction. It can be used without discomfort. This brace restricts neck movements in both the sagittal and coronal planes (figures 2 and 3). There is no tension on sutures applied for approximating the chin to the chest wall.

The first patient who used this brace was a 27-year-old male with tracheal stenosis due to prolonged intubation. He wore the brace after surgery with adequate levels of comfort and fit. The brace maintained the desirable neck position for one week after which the patient was discharged without any complications. Subsequently, we have used this brace for ten additional patients with no observed complications. Currently, we have decided to use it for any patient who undergoes tracheal reconstruction. 

## Discussion

Tracheal resection and end-to-end anastomosis are accompanied by a significant tension at the anastomosis site. Increasing the length of the tracheal resection will result in a progressive rise in tension. The safe limit of resection to avoid excess tension and anastomotic failure is estimated to be approximately 4.5 cm.^2^ Release of surrounding tissues and maintaining the neck in a proper position (i.e., full flexion) are measures to prevent tension-induced anastomotic failure. As previously mentioned, this position is established by a suture placed between the chin and anterior part of the upper chest. To diminish its annoying use and establish additional support for reinforcing neck flexion, a 100 cm fiberglass-made orthosis has been introduced by Mueller et al. in 2004.^3^ Another innovative tool was invented by an Indian group in which a poly ethylene made brace was used for flexion.^4^ In another study by Imai et al. in 2013, a halo vest immobilization was performed on patients with reconstruction that attached the device to the skull by pins.^5^

The brace that we have designed for our patients seems to be more practical due to its increased comfort and fit by using strip bands and additional extended power to prevent any movement in both the sagittal and coronal planes. We suppose that this brace does not allow for lateral neck bending or rotation, both of which are not sufficiently prevented in the previously designed orthosis by Muller. Our washable brace is lightweight and of low cost. It can be safely used in patients who are not cooperative enough or those with mental problems, both of which contribute to the high chances for disrupting the supporting suture. Analyses of blood gas levels post-surgery have shown no respiratory compromise in patients during use of the brace. Patients can walk and perform their normal daily activities.

Comparing to the brace that was introduced by Imai in Japan our brace application is noninvasive. Additionally, due to the presence of the anterior and posterior segments that cover the chest, the brace has decreased impact on respiration compared with the Indian model. Our device is made of fiberglass and is fenestrated so it causes less sweating with decreased chances for pressure ulcer formation. 
